# Synergistic effects of microwave pretreatment and sous-vide cooking on flavor modulation and taste precursor dynamics in chicken breast

**DOI:** 10.1016/j.fochx.2026.104013

**Published:** 2026-05-20

**Authors:** Shuqiang Zhang, Yungang Cao, Min Li, Zhijian Wang, Bin Yu, Haiteng Tao, Wei Gao, Jianpeng Li, Zheng Zhang, Haibo Zhao, Zhengzong Wu, Bo Cui

**Affiliations:** a Shandong Key Laboratory of Healthy Food Resources Exploration and Creation, and School of Food Science and Engineering, Qilu University of Technology, Shandong Academy of Sciences, Jinan 250353, China; b School of Food Science and Engineering, Shaanxi University of Science & Technology, Xi'an 710021, China; c Shandong Delisi Food Co., Ltd., Weifang 262216, China; d Shandong Huifa Food Co., Ltd., Weifang 262200, China; e Weifang Meicheng Foodstuffs Co., Ltd., Weifang 261051, China

**Keywords:** Microwave pretreatment, Sous-vide cooking, Taste precursors, Volatile compounds, Flavor modulation

## Abstract

Sous-vide cooking ensures tenderness but often results in limited flavor development. This study investigated microwave pretreatment as a rapid strategy to enhance flavor formation prior to sous-vide processing of chicken breast. The combined treatment significantly increased free amino acids (FAA; 19.51 mg g^−1^) and umami-related nucleotides, with glutamic acid identified as the dominant contributor. Gas chromatography–mass spectrometry (GC–MS) identified 57 volatile compounds, and the abundance of key odorants increased by 28%–35% (*p* < 0.05) following microwave–sous-vide treatment. Two-way ANOVA revealed significant synergistic effects (*p* < 0.01) between microwave duration and sous-vide conditions on both precursor liberation and volatile formation. Overall, microwave-assisted sous-vide processing enhances flavor while retaining the benefits of mild thermal treatment, providing a practical approach for producing high-quality poultry with superior flavor attributes.

## Graphical abstract

**Figure:**
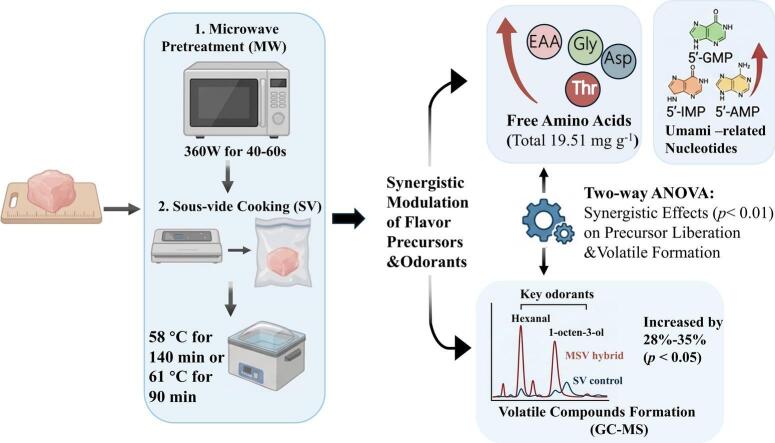

## 1 Introduction

Chicken breast has emerged as a predominant poultry product in global consumption, attributable to its high protein content, low fat composition, and the lack of religious dietary restrictions (Cao et al., 2021). While thermal processing is fundamentally required to eliminate pathogens and ensure food safety, it is equally critical for transforming the unpalatable sensory attributes of raw meat into a desirable consumer experience (Wołoszyn et al., 2020). Beyond basic edibility, cooking triggers complex chemical reactions that generate the diverse volatile and non-volatile compounds responsible for a product's characteristic aroma, texture, and mouthfeel (You et al., 2023). Flavor quality serves not only a critical determinant of consumer acceptance but also a central objective in processing optimization (Jayasena et al., 2013). Tuorila et al. (1995) reported that fat interacted in complex ways with volatile and nonvolatile ingredients, thus affecting the timing of flavor release in a product. The development of meat flavor arises from intricate biochemical pathways, including Maillard reactions, lipid oxidation, and nucleotide degradation (Shahidi, 1998). Consequently, precise control of thermal processing parameters plays a key role in modulating the conversion efficiency of these flavor precursors.

Contemporary research on poultry flavor modulation has predominantly focused on single heating technologies, including microwave irradiation (Domínguez et al., 2014) or vacuum low-temperature cooking (sous-vide) (Przybylski et al., 2021). Sous-vide is recognized for its gentle temperature control and potential for tenderness and nutrient retention; however, it often requires extended cooking times to achieve sufficient precursor liberation and aroma complexity (Aguilera, 2018). In contrast, microwave heating provides rapid volumetric heating through dipole rotation and ionic conduction (Cullen et al., 2012), but its non-uniform energy distribution can cause local overheating and undesirable reaction products (Vadivambal & Jayas, 2010). These limitations suggest that integrating the rapid initiation advantage of microwave pretreatment with the mild and controllable characteristics of sous-vide heating may offer a practical route to enhance flavor formation while reducing undesirable reactions. Nevertheless, systematic evidence of synergistic effects and the underlying regulatory mechanisms—particularly regarding taste precursors and volatile formation—remains limited.

To address this gap, the present study proposes a hybrid strategy of microwave pretreatment followed by sous-vide cooking, designed to couple rapid structural activation with controlled low-temperature conversion and retention of key flavor components. Prior work has shown that combining microwave energy with conventional heating can enhance heating uniformity, improve energy efficiency, and modulate oxidation-related pathways (Datta & Rakesh, 2013; Jantaranikorn et al., 2023), suggesting potential for directing polyunsaturated fatty-acid oxidation toward odor-active products under controlled conditions.

The primary objective of this study was to evaluate the impact of this hybrid microwave–sous-vide process on the comprehensive flavor profile of chicken breast. To achieve this, a multidimensional analytical framework was employed: high-performance liquid chromatography (HPLC) for the quantification of umami-contributing nucleotides; gas chromatography–mass spectrometry (GC–MS) for the characterization of volatile organic compounds; and electronic nose (e-nose) technology for holistic aroma profiling. The results of this study are intended to provide both mechanistic clarity and practical guidance for the development of poultry processing technologies that strike an optimal balance between nutrient retention and sensory excellence.

## 2 Materials and methods

### 2.1 Experimental materials

Frozen chicken breasts were procured from a local supermarket (white-feathered broilers; Chunxue Food Co., Ltd., China). These breasts were allocated to three independent biological batches (20 pieces per batch) to ensure genuine biological replicates. To minimize individual variation, each batch was processed independently. Samples were thawed at 4 °C for 24 h, after which tendons and connective tissue were removed. The chicken breasts were cut into 3 × 3 × 1 cm cubes and randomly assigned to experimental groups. Each treatment comprised three replicates, with meat cubes from different independent batches used within each replicate group. This design ensured experimental units were mutually independent, avoiding potential pseudo-replication. Initial color values (L*: 53.71 ± 1.90, a*: 7.98 ± 1.30, b*: 15.67 ± 0.32) were recorded using a chroma meter (CR-400, Konica Minolta, Japan).

### 2.2 Experimental design

Treatment groups and coding are summarized in Table S1.

Briefly, the study comprised a 3 × 2 factorial MSV design (microwave pretreatment time + sous-vide condition) plus single-process controls:

(i) Sous-vide controls (SV): SV1(T: 58 °C, t: 140 min) and SV2 (T: 61 °C, t: 90 min).

(ii) Microwave-only controls (M): M1, M2, M3 (P:1080 W for t: 40, 50, 60 s, respectively).

(iii) Microwave pretreatment + sous-vide (MSV): Microwave pretreatment was performed at P: 360 W for 40 s (M1SV), 50 s (M2SV), or 60 s (M3SV), followed by sous-vide at either T: 58 °C for 140 min (SV1) or T: 61 °C for 90 min (SV2), yielding M1SV1–M3SV1 and M1SV2–M3SV2.

For sous-vide processing, samples were placed in nylon–polyethylene (PE) bags and vacuum sealed (−1 MPa; vacuum time 40 s; sealing time 2 s; cooling time 2 s), then cooked in a constant-temperature water bath (T: 58 °C, t: 140 min or T: 61 °C, t: 90 min). For microwave pretreatment, bagged samples were microwaved at 360 W for 40–60 s before being vacuum sealed and sous-vide cooked. For microwave-only controls, samples were heated directly in a microwave oven at P: 1080 W for 40–60 s. After heating, samples were cooled to room temperature and stored at −38 °C until analysis.

### 2.3 Physical properties

#### 2.3.1 Color measurement

A colorimeter (CR-400, Konica Minolta, Tokyo, Japan) was calibrated using a white ceramic tile, and the values of L* (lightness/darkness), a* (redness/greenness), and b* (yellowness/blueness) were obtained. The surface color of each sample was measured at eight random locations after standardization.

#### 2.3.2 Flavor-presenting nucleotide analysis

Nucleotides were extracted from minced samples (10 g) using 30 mL of ice-cold perchloric acid (5%, *v*/v). The mixture was subjected to ultrasonication for 10 min, followed by centrifugation at 12,000 ×*g* for 10 min at 4 °C. The resulting filtrates were adjusted to pH 6.5 using 0.1 mol L^−1^ NaOH and filtered through a 0.22-μm membrane. The filtrate was analyzed using an HPLC system (LC-20 A, Shimadzu, Kyoto, Japan) equipped with a UV-VIS detector (SPD-20 A). Separation was performed on a Sunfire C18 column (4.6 mm × 255 mm, 5 μm). The mobile phase consisted of a phosphate buffer and methanol (95:5, v/v), with detection monitored at 254 nm (Yue et al., 2016).

#### 2.3.3 Free amino acid analysis

Freeze-dried samples were hydrolyzed and derivatized. All meat samples were freeze-dried and subsequently ground into powder. One gram of powder was accurately weighed, added to 40 mL of deionized water, and sonicated for 20 min (80 Hz, 350 W). The volume was then diluted to 50 mL. Next, add 4 mL of 10% sulfosalicylic acid to 4 mL of the solution, shake vigorously, and allow to stand for 2 h to precipitate the proteins. After filtration, the mixture was filtered again and separately added to 2 mL of 1% EDTA-Na and 0.06 mol L^−1^ hydrochloric acid. After shaking for 5 min and centrifuging (15,000 rpm, 10 min), analyze 1 mL of the supernatant using a Hitachi L-8900 high-speed amino acid analyzer (Li et al., 2022).

#### 2.3.4 Taste activity value (TAV) determination

The TAV of each tastant was calculated as the ratio of its absolute concentration to its taste threshold value (Bai et al., 2022; Zheng et al., 2025). Generally, compounds with TAV > 1 are considered active contributors to food flavor.

#### 2.3.5 Equivalent umami concentration (EUC) determination

The EUC represents the concentration of monosodium glutamate, equivalent to the umami intensity produced by the synergistic effect of umami amino acids and 5′-nucleotides. The EUC was calculated using the following equation (Zheng et al., 2025).

$$Y=\sum a_{i}b_{i}+1218\left(\sum a_{i}b_{i}\right)\left(\sum a_{j}b_{j}\right)$$

#### 2.3.6 Thiobarbituric acid (TBA) value

The TBA value was determined following the method of Lan et al. (2016). Minced samples of 10 g were mixed with 25 mL of 20% (*w*/*v*) trichloroacetic acid solution and homogenized using a tissue homogenizer for 30 s. The mixture was centrifuged at 5500 rpm for 15 min at 4 °C, and the supernatant was filtered twice (Allegra 64R, Thunder Magnetic Instrument Co., Ltd., Shanghai, China). A 2 mL aliquot of the filtrate was mixed with 2 mL of 0.02 mol L^−1^ TBA solution in a test tube and incubated at 80 °C for 20 min. After incubation, the mixture was cooled in an ice bath for 10 min. Absorbance was measured at 532 nm using a spectrophotometer (UV2550, Spectrum General Technology Co., Ltd., Beijing, China), with water as the blank. Control samples were prepared and analyzed using the same protocol as the experimental groups.

#### 2.3.7 Fatty acid analysis

After lyophilization, all meat samples were ground into powder. A 1.0 g aliquot of the powder was accurately weighed and transferred to a conical flask for solvent extraction. Subsequently, 30 mL of extraction solution (chloroform: methanol = 2:1, *v*/v) was added. The mixture was homogenized for 1 min using a tissue homogenizer, filtered, and mixed with 30 mL of 1 mol L^−1^ KCl solution. The samples were refrigerated at 4 °C overnight.

For methyl esterification, the lower fatty acid extract was dried at 60 °C and dissolved in 5 mL of hexane. Subsequently, 5 mL of KOH–methanol solution was added, and the mixture was incubated at 50 °C for 1 h. After methyl esterification, 10 mL of 5% HCl was added, followed by 5 mL of hexane. Then, the mixture was left to stand for 15 min, after which the upper layer was collected and diluted to a final volume of 10 mL.

Fatty acid analysis was performed using a Thermo Fisher TSQ8000 Evo gas chromatograph equipped with a flame ionization detector and a split-flow injector. The column used was an SP-2560 capillary column (30 cm × 0.25 mm × 0.20 μm). The temperature condition was as follows: initial temperature of 50 °C held for 3 min, ramped at 10 °C/min to 220 °C, and held for 20 min. Detector and injector temperatures were maintained at 250 °C. Samples were injected at a split ratio of 10:1.

Fatty acids were characterized by comparing the detected peaks with the retention times of a fatty acid standard mixture (47,015-U, Sigma-Aldrich, MO, USA).

#### 2.3.8 Determination of volatile flavors

The volatile compounds were analyzed using a GC–MS system (Thermo Fisher, TSQ 8000 Evo, Guangzhou, China) equipped with a silica column (30 m × 0.25 mm × 0.25 μm, Nonpolar). A 5 g portion of uniformly chopped chicken meat sample was placed in a 20 mL headspace vial for solid-phase microextraction (SPME). Volatiles were extracted using an SPME fiber (50/30 μm CAR/PDMS extraction head, Supelco, Bellefonte, USA), following the procedure previously described (H. Wang et al., 2022). Samples were equilibrated for 10 min at 60 °C, after which the fiber was exposed to the headspace for 40 min at 60 °C.

After extraction, the SPME fiber was transferred into the gas chromatograph inlet (250 °C) for desorption for 3 min. Separation of the volatile compounds was done on a fused silica column. The instrument temperature was programmed as follows: 45 °C for 3 min, increased to 150 °C at 4 °C/min, then to 250 °C at 8 °C/min, and held for 6 min. Mass spectrometry was performed in the range 35–500 amu using electron collision ionization mode with helium as the carrier gas at a flow rate of 1.0 mL min^−1^ and in the 70 eV electron collision ionization mode. The ion source was maintained at 200 °C. Volatile compounds were identified by GC–MS analysis based on reference mass spectra from computer-matched NIST11 and NIST11s mass spectral libraries.

#### 2.3.9 *E*-nose analysis

The volatile flavor profiles were further analyzed using a PEN3 electronic nose system (Airsense Analytics GmbH, Schwerin, Germany). The system is equipped with 10 different metal oxide sensors (Table S2). Data processing was performed using WinMuster software associated with the equipment. Before analysis, 2 g of each sample was equilibrated at 60 °C for 10 min. This was followed by analysis at a carrier gas flow rate of 400 mL min^−1^. Data analysis was performed using PCA and PLS-DA with WinMuster software (version 1.6, Airsense Analytics GmbH, Schwerin, Germany).

#### 2.3.10 Statistical analysis

Data are presented as mean ± SD with ≥3 independent biological replicates. Normality and variance homogeneity were assessed using Shapiro–Wilk and Levene's tests (*p* > 0.05). For the microwave pretreatment + sous-vide set (M1SV1–M3SV2), a two-way ANOVA model was used with microwave time (40/50/60 s), sous-vide condition (58 °C/140 min; 61 °C/90 min), and their interaction to evaluate synergy. For comparisons across all groups (M, SV, MSV), one-way ANOVA with Duncan's multiple range test (*p* < 0.05) was used. Statistical analyses were performed using SPSS Statistics 24.0 (IBM Corp., Armonk, NY, USA). Figures were generated using Origin 2018b (Origin Lab, Northampton, MA, USA).

## 3 Results and discussion

### 3.1 Color

Lightness (L*) reflects the whiteness and brightness of the samples, with higher values indicating a brighter surface of the chicken breast. The redness index (a*) correlates with the freshness of the chicken breast meat and aligns with consumers' preference for more intense red coloration (Moyo et al., 2020). As shown in Table 1, microwave–sous-vide processing increased L* and decreased a* compared with controls, with the maximum L* (83.27) observed for 360 W pretreatment (40 s) followed by 58 °C/140 min sous-vide (M1SV1). This trend is consistent with the findings of Modzelewska-Kapituła et al. (2023), who reported that rapid protein denaturation under electromagnetic-assisted heating leads to increased light scattering at the meat surface. These changes may be explained by moisture redistribution that alters light penetration/reflectance and protein denaturation that increases light scattering (Christensen et al., 2011; Mar et al., 2013). With increasing microwave pretreatment time, redness loss was more pronounced, consistent with accelerated myoglobin denaturation. In addition, the higher-temperature sous-vide condition (61 °C) produced higher b* values across treatments, indicating a temperature-driven yellowing effect during cooking.

**Table 1:** Surface color parameters (L*, a*, and b*) of chicken breast subjected to different thermal treatments.

samples	L*	a*	b*
M1	73.24 ± 0.73^g^	2.91 ± 0.02^g^	15.76 ± 0.11^a^
M2	75.78 ± 0.34^f^	3.92 ± 0.01^d^	15.21 ± 0.06^b^
M3	77.85 ± 0.06^e^	3.37 ± 0.14^e^	13.67 ± 0.04^d^
M1SV1	83.27 ± 0.09^a^	3.06 ± 0.05^f^	13.23 ± 0.04^f^
M2SV1	81.62 ± 0.09^cd^	3.43 ± 0.02^e^	12.46 ± 0.04^h^
M3SV1	82.08 ± 0.09^c^	3.37 ± 0.12^e^	13.43 ± 0.09^e^
SV1	81.41 ± 0.02^d^	4.21 ± 0.06^c^	13.11 ± 0.04^fg^
M1SV2	82.72 ± 0.04^b^	4.36 ± 0.04^b^	14.61 ± 0.09^c^
M2SV2	81.68 ± 0.05^cd^	3.46 ± 0.05^e^	12.99 ± 0.99^g^
M3SV2	81.23 ± 0.06^d^	4.75 ± 0.05^a^	13.57 ± 0.04^d^
SV2	82.61 ± 0.04^b^	4.13 ± 0.03^c^	13.41 ± 0.04^e^

Values are mean ± SD (*n* = 3). Different superscript letters within the same column indicate significant differences among treatments (*p* < 0.05). Treatment codes are defined in Table S1.

### 3.2 Flavor-presenting nucleotides analysis

**Fig. 1:**
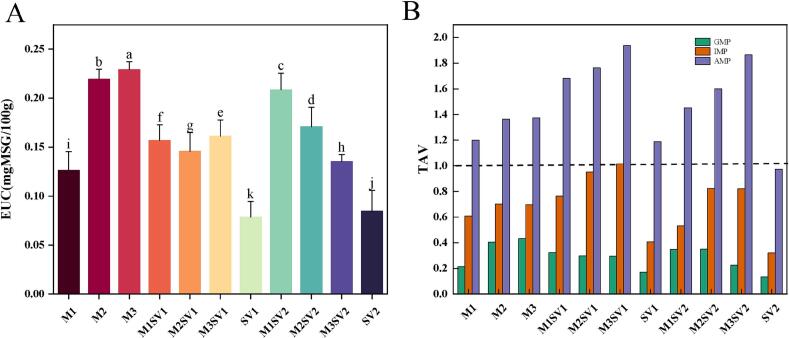
Equivalent umami concentration (EUC; g MSG/100 g) (A) and taste activity values (TAVs) of umami-related nucleotides (B) in chicken breast subjected to different thermal treatments (M1–M3, microwave-only; SV1–SV2, sous-vide controls; M1SV1–M3SV2, microwave pretreatment + sous-vide). Bars represent mean ± SD (*n* = 3). Different letters in (A) indicate significant differences (*P* < 0.05). The dashed line in (B) denotes TAV = 1.

Nucleotides, particularly guanosine 5′-monophosphate (5’-GMP), inosine 5′-monophosphate (5’-IMP), and adenosine 5′-monophosphate (5’-AMP), are core contributors to umami perception in cooked meat. Microwave–sous-vide treatment significantly increased GMP, IMP, and AMP concentrations relative to sous-vide controls (*P* < 0.05) (Przybylski et al., 2021). As summarized in Table 2, the 360 W–60 s pretreatment combined with 58 °C/140 min sous-vide (M3SV1) yielded the highest nucleotide enrichment. With marked increases in AMP and IMP relative to SV controls. EUC values (Fig. 1A) further supported enhanced umami intensity, while TAV analysis (Fig. 1B) indicated that AMP remained taste-active (TAV > 1) across microwave-treated groups. Under the 61 °C condition, IMP and AMP exhibited a non-linear (biphasic) response across microwave times, suggesting concurrent formation and degradation reactions during processing. Overall, these results indicate that microwave pretreatment can shift nucleotide-related pathways in a direction favorable to umami enhancement when coupled with controlled sous-vide heating.

**Table 2:** Umami-related nucleotide contents (5′-GMP, 5′-IMP, and 5′-AMP; μg g^−1^) and lipid oxidation index (TBARS; expressed as mg MDA kg^−1^) of chicken breast under different thermal treatments.

samples	M1	M2	M3	M1SV1	M2SV1	M3SV1	SV1	M1SV2	M2SV2	M3SV2	SV2
GMP	1.73 ± 0.91^h^	3.29 ± 0.25^b^	3.51 ± 0.29^a^	2.63 ± 0.20^d^	2.42 ± 0.06^e^	2.40 ± 0.57^f^	1.39 ± 0.21^i^	2.83 ± 0.03^c^	2.84 ± 0.21^c^	1.83 ± 0.38^g^	1.10 ± 0.42^j^
IMP	15.53 ± 0.30^f^	17.93 ± 0.51^e^	17.80 ± 0.35^e^	19.47 ± 0.15^d^	24.30 ± 0.19^b^	25.91 ± 0.21^a^	10.39 ± 0.15^h^	13.58 ± 0.28^g^	20.99 ± 0.15^c^	20.96 ± 0.02^c^	8.21 ± 0.24^i^
AMP	30.65 ± 0.54^h^	34.83 ± 0.79^g^	35.09 ± 0.35^g^	42.97 ± 1.18^d^	45.03 ± 0.48^c^	49.46 ± 0.12^a^	30.36 ± 0.97^h^	37.07 ± 0.35^f^	40.86 ± 0.01^e^	47.62 ± 0.46^b^	24.87 ± 0.83^i^
TBARS	1.48 ± 0.07^e^	2.31 ± 0.02^a^	1.00 ± 0.09^g^	1.29 ± 0.09^f^	1.03 ± 0.02^g^	1.77 ± 0.10^c^	0.65 ± 0.03^h^	2.18 ± 0.02^b^	1.35 ± 0.01^f^	2.20 ± 0.04^b^	1.63 ± 0.02^d^

Values are mean ± SD (n = 3). Different superscript letters within the same row indicate significant differences among treatments (*p* < 0.05). Treatment codes are defined in Table S1.

### 3.3 Analysis of free amino acids

Free amino acids (FAAs) are important flavor precursors and nutritional indicators. (Eman Sh. Al et al., 2016). As summarized in Table 3, total FAA content varied significantly among different treatments (9.55–19.51 mg g^−1^), and the highest value occurred for 360 W–60 s pretreatment followed by 61 °C/90 min sous-vide (M3SV2), representing a 53.5% increase compared with SV2. Glutamic acid exhibited the highest taste contribution, indicating that FAA enrichment translated into meaningful sensory improvements rather than merely an increase in total concentration. Under the 360 W pretreatment, FAA content increased with pretreatment duration, which may reflect enhanced protein unfolding and hydrolysis that promotes FAA liberation and accumulation (Guo et al., 2021). In addition, the EAA/NEAA ratio increased significantly at 61 °C (*p* < 0.05), suggesting improved amino-acid balance under that cooking condition (Hurst, 2025). Importantly, two-way ANOVA demonstrated a significant interaction between microwave time and sous-vide condition regarding the FAA content and nucleotide enrichment (*p* < 0.01, Table S3). This confirms that the combination of microwave pretreatment and sous-vide cooking exerts a synergistic effect rather than a mere additive effect, optimizing the flavor profile beyond what either treatment could achieve independently.

**Table 3:** Free amino acid composition (mg g^−1^) of chicken breast after different thermal treatments, grouped by taste attributes, with summary indices (total FAAs, total essential amino acids (EAA), and E/NE ratio).

amino acids	Flavor	M1	M2	M3	M1SV1	M2SV1	M3SV1	SV1	M1SV2	M2SV2	M3SV2	SV2
Asp	umami	0.96 ± 0.02^a^	0.77 ± 0.03^d^	0.59 ± 0.00^f^	0.77 ± 0.02^d^	0.53 ± 0.01^g^	0.75 ± 0.01^d^	0.64 ± 0.02^e^	0.87 ± 0.01^b^	0.77 ± 0.02^d^	0.83 ± 0.02^c^	0.66 ± 0.01^e^
Thr	salty	1.26 ± 0.00^h^	1.35 ± 0.00^g^	1.45 ± 0.01^f^	1.61 ± 0.02^de^	1.52 ± 0.02^f^	2.17 ± 0.02^b^	1.67 ± 0.02^d^	1.58 ± 0.01^ef^	1.67 ± 0.01^d^	2.56 ± 0.00^a^	1.76 ± 0.01^c^
Glu	umami	0.11 ± 0.00^d^	0.12 ± 0.00^c^	0.13 ± 0.02^b^	0.10 ± 0.00^e^	0.12 ± 0.00^c^	0.14 ± 0.00^a^	0.10 ± 0.00^e^	0.13 ± 0.00^b^	0.12 ± 0.00^c^	0.14 ± 0.00^a^	0.11 ± 0.00^d^
Gly	salty	1.63 ± 0.06^g^	1.80 ± 0.12^f^	1.87 ± 0.08^ef^	1.80 ± 0.01^f^	2.05 ± 0.02^h^	2.89 ± 0.01^b^	1.91 ± 0.01^e^	2.02 ± 0.01^d^	2.16 ± 0.01^c^	3.38 ± 0.00^a^	2.17 ± 0.01^c^
Cys	salty	0.33 ± 0.01^fg^	0.38 ± 0.01^bc^	0.37 ± 0.00^c^	0.32 ± 0.02^g^	0.34 ± 0.02^de^	0.45 ± 0.01^a^	0.35 ± 0.00^d^	0.38 ± 0.00^bc^	0.39 ± 0.02^b^	0.45 ± 0.00^a^	0.37 ± 0.01^c^
Val	bitter	0.72 ± 0.03^g^	0.80 ± 0.06^ef^	0.84 ± 0.04^e^	0.66 ± 0.04^g^	0.93 ± 0.05^d^	1.29 ± 0.01^b^	0.78 ± 0.00^f^	0.94 ± 0.00^d^	0.91 ± 0.05^d^	1.52 ± 0.00^a^	1.01 ± 0.01^c^
Met	bitter	0.36 ± 0.00^d^	0.48 ± 0.02^c^	0.50 ± 0.01^c^	0.39 ± 0.08^d^	0.51 ± 0.08^c^	0.78 ± 0.01^a^	0.48 ± 0.00^c^	0.52 ± 0.01^c^	0.50 ± 0.10^c^	0.80 ± 0.02^a^	0.63 ± 0.03^b^
Ile	bitter	0.38 ± 0.02^f^	0.43 ± 0.04^e^	0.45 ± 0.02^e^	0.37 ± 0.01^f^	0.53 ± 0.01^d^	0.76 ± 0.01^b^	0.42 ± 0.00^e^	0.52 ± 0.01^d^	0.52 ± 0.01^d^	0.89 ± 0.00^a^	0.57 ± 0.01^c^
Leu	bitter	0.66 ± 0.04^h^	0.77 ± 0.08^g^	0.82 ± 0.05^f^	0.67 ± 0.01^h^	0.95 ± 0.01^d^	1.33 ± 0.01^b^	0.75 ± 0.01^g^	0.89 ± 0.01^e^	0.91 ± 0.00^de^	1.60 ± 0.03^a^	1.02 ± 0.02^c^
Tyr	bitter	0.61 ± 0.04^f^	0.66 ± 0.06^e^	0.73 ± 0.04^d^	0.64 ± 0.01^ef^	0.73 ± 0.01^d^	1.13 ± 0.01^b^	0.69 ± 0.00^e^	0.74 ± 0.01^d^	0.80 ± 0.01^c^	1.25 ± 0.07^a^	0.81 ± 0.05^c^
Phe	bitter	0.50 ± 0.03^f^	0.57 ± 0.05^e^	0.61 ± 0.04^de^	0.51 ± 0.01^f^	0.66 ± 0.01^cd^	0.96 ± 0.01^b^	0.57 ± 0.01^e^	0.62 ± 0.01^de^	0.68 ± 0.02^c^	1.02 ± 0.08^a^	0.64 ± 0.06^cd^
Lys	bitter	0.90 ± 0.03^g^	1.19 ± 0.06^e^	1.26 ± 0.06^d^	0.67 ± 0.01^h^	1.27 ± 0.02^d^	1.98 ± 0.01^b^	0.85 ± 0.01^g^	1.09 ± 0.08^f^	1.33 ± 0.01^c^	2.45 ± 0.02^a^	1.31 ± 0.04^cd^
His	bitter	1.55 ± 0.00^b^	1.47 ± 0.01^c^	1.40 ± 0.02^d^	0.37 ± 0.01^j^	0.49 ± 0.01^h^	0.64 ± 0.00^g^	0.45 ± 0.01^i^	1.96 ± 0.02^a^	0.41 ± 0.03^i^	1.01 ± 0.02^e^	0.66 ± 0.01^f^
Arg	bitter	0.86 ± 0.04^e^	1.00 ± 0.07^d^	0.99 ± 0.05^d^	0.67 ± 0.01^g^	0.90 ± 0.00^e^	1.43 ± 0.02^b^	0.76 ± 0.01^f^	1.58 ± 0.07^a^	1.10 ± 0.00^c^	1.61 ± 0.01^a^	0.99 ± 0.00^d^
Total free amino acid content		10.83 ± 0.30^h^	11.78 ± 0.41^fg^	12.01 ± 0.44^ef^	9.55 ± 0.26^i^	11.40 ± 0.27^g^	16.70 ± 0.14^b^	10.43 ± 0.10^h^	13.83 ± 0.25^c^	12.28 ± 0.29^de^	19.51 ± 0.27^a^	12.71 ± 0.27^d^
Total Essential Amino Acids (EAA)		4.51 ± 0.12^h^	5.26 ± 0.19^g^	5.62 ± 0.17^f^	4.61 ± 0.07^h^	5.96 ± 0.08^e^	8.65 ± 0.04^b^	5.20 ± 0.02^g^	5.75 ± 0.03^ef^	6.13 ± 0.08^d^	10.08 ± 0.11^a^	6.48 ± 0.10^c^
E/NE		0.73	0.80	0.87	0.93	1.09	1.07	0.99	0.71	0.99	1.06	1.04

Values are mean ± SD (n = 3). Different superscript letters within the same row indicate significant differences among treatments (*p* < 0.05). Treatment codes are defined in Table S1.

### 3.4 Dynamic balance regulation of flavor precursors

Flavor nucleotides and free amino acids (FAAs) are critical for umami perception and are highly sensitive to thermal processing (Zhang et al., 2023). In the present study, the hybrid microwave–sous-vide (MSV) treatment increased 5’-IMP content by 25% relative to the control. This finding is consistent with the observations of Xu et al. (2026), who suggested that moderate electromagnetic heating can promote the degradation of ATP into umami-contributing nucleotides. Notably, the MSV treatment achieved higher retention rates than those observed by Niu et al. (2023) using conventional boiling. This discrepancy is likely attributable to the “matrix-retention effect” facilitated by the vacuum-sealed environment of the sous-vide phase. By eliminating the cooking medium (water) and maintaining structural integrity, the vacuum seal prevents the convective leaching of water-soluble nucleotides, thereby concentrating flavor precursors within the meat matrix rather than losing them to the surrounding environment.

Microwave pretreatment (360 W, 60 s) markedly increased nucleotide accumulation. For instance, in the 61 °C/90 min condition, 5′-IMP (20.96 mg (100 g)^−1^) and 5′-GMP (1.83 mg (100 g)^−1^) increased by 155% and 66%, respectively, relative to sous-vide at the same cooking condition. This enrichment may be associated with accelerated conversion along AMP–IMP-related pathways under microwave-induced structural/enzymatic activation (Yue et al., 2016). Additionally, vacuum low-temperature processing (61 °C for 90 min) effectively reduced the degradation of 5’-IMP by inhibiting 5′-nucleotidase (5’-NT) activity (Mottram, 1998).

Consistent with this coupling, total FAA content increased strongly in the same optimal combined condition (from 12.71 to 19.51 mg g^−1^). Notably, glutamic acid (Glu) and aspartic acid (Asp) increased by 118% and 96%, respectively, which could be attributed to microwave-induced protein denaturation and vacuum-assisted activation (Wall et al., 2019). To further elucidate the potential mechanism, a Pearson correlation analysis was performed (Fig. S1). The heatmap reveals a strong positive correlation (*r* > 0.85, *p* < 0.05) between microwave pretreatment time and the accumulation of 5’-IMP and total FAAs. These findings support a coordinated regulatory effect of the hybrid process on precursor liberation, contributing to enhanced umami potential (Fig. 1).

### 3.5 Lipid oxidation

TBA is widely used as an indicator of secondary lipid oxidation products in meat systems (Liu et al., 2025). Thermal conditions affect oxidation kinetics, and microwave pretreatment can intensify early-stage oxidation by rapidly increasing the temperature and accelerating oxygen- and radical-mediated reactions. At the same time, aldehydes generated from oxidation are chemically reactive and may form Schiff bases with amino compounds (Hammud et al., 2015), potentially influencing apparent TBA responses by reducing the pool of freely reactive aldehydes. Therefore, between-group differences in TBA values likely reflect the balance between aldehyde generation and subsequent conversion reactions. This interpretation is consistent with our GC–MS results showing elevated aldehyde abundance in microwave-pretreated samples (Section 3.7).

### 3.6 Differential analysis of free fatty acid content

The major free fatty acids included C18:2, C18:1, and C16:0 across all treatments (Table 4). Microwave processing increased the proportion of monounsaturated fatty acid (MUFA), peaking at 43.26%, which may have nutritional relevance given reported associations between MUFA intake and improved lipid profiles (Kien et al., 2014; Li et al., 2016). In contrast, sous-vide cooking alone reduced MUFA and polyunsaturated fatty acids (PUFA) levels, potentially due to the migration of hydrolyzed fats into exudate/broth. This result is consistent with the previous findings (Silva et al., 2016; Yu et al., 2021). Microwave pretreatment before sous-vide altered the fatty acid (FA) composition in a time-dependent manner, increasing free fatty-acid levels and reducing C14:0 relative to controls. These results indicate that microwave pretreatment reshapes the substrate pool for lipid-derived aroma formation and may also modify nutritional lipid profiles.

**Table 4:** Fatty acid composition of chicken breast after different thermal treatments (μg g^−1^), including totals for saturated (SFA), monounsaturated (MUFA), polyunsaturated (PUFA), and essential fatty acids (EFA).

fatty acids	M1	M2	M3	M1SV1	M2SV1	M3SV1	SV1	M1SV2	M2SV2	M3SV2	SV2
C12:0	0.03 ± 0.00^e^	0.05 ± 0.01^c^	0.03 ± 0.00^e^	0.03 ± 0.00^e^	0.05 ± 0.00^c^	0.07 ± 0.00^b^	0.02 ± 0.00^f^	0.03 ± 0.00^e^	0.04 ± 0.01^d^	0.11 ± 0.00^a^	0.03 ± 0.00^e^
C14:0	0.61 ± 0.02^e^	0.80 ± 0.16^d^	0.70 ± 0.01^de^	0.60 ± 0.01^e^	1.05 ± 0.01^c^	1.34 ± 0.03^b^	0.48 ± 0.00^f^	0.70 ± 0.01^de^	0.79 ± 0.13^d^	2.01 ± 0.05^a^	0.72 ± 0.05^d^
C14:1	0.62 ± 0.02^a^	0.08 ± 0.02^de^	0.08 ± 0.01^de^	0.07 ± 0.01^e^	0.10 ± 0.01^d^	0.14 ± 0.03^c^	0.05 ± 0.02^f^	0.07 ± 0.03^e^	0.08 ± 0.01^de^	0.23 ± 0.01^b^	0.08 ± 0.00^de^
C15:0	0.10 ± 0.00^ef^	0.13 ± 0.03^d^	0.07 ± 0.00^g^	0.10 ± 0.00^ef^	0.18 ± 0.01^c^	0.23 ± 0.00^b^	0.08 ± 0.00^f^	0.11 ± 0.00^e^	0.13 ± 0.02^d^	0.34 ± 0.01^a^	0.13 ± 0.01^d^
C16:0	12.21 ± 0.11^f^	15.78 ± 0.76^d^	12.35 ± 0.13^f^	11.75 ± 0.08^g^	18.02 ± 0.25^c^	20.30 ± 0.48^b^	10.19 ± 0.05^h^	13.13 ± 0.17^e^	15.50 ± 0.43^d^	26.94 ± 0.55^a^	13.45 ± 0.73^e^
C16:1	3.13 ± 0.05^f^	3.77 ± 0.41^e^	24.75 ± 0.26^a^	2.95 ± 0.02^g^	5.12 ± 0.08^d^	6.87 ± 0.33^c^	2.46 ± 0.01^h^	3.19 ± 0.05^f^	3.54 ± 0.72^ef^	9.73 ± 0.19^b^	3.51 ± 0.26^ef^
C17:0	0.19 ± 0.01^e^	0.25 ± 0.05^d^	0.23 ± 0.01^d^	0.19 ± 0.00^e^	0.36 ± 0.01^c^	0.46 ± 0.00^b^	0.18 ± 0.00^e^	0.22 ± 0.03^de^	0.38 ± 0.09^c^	0.68 ± 0.01^a^	0.27 ± 0.02^d^
C18:0	11.27 ± 0.16^f^	14.94 ± 0.88^d^	11.46 ± 0.12^f^	11.12 ± 0.15^f^	17.79 ± 0.45^c^	19.76 ± 0.41^b^	10.15 ± 0.01^g^	12.77 ± 0.16^e^	15.38 ± 0.33^d^	26.90 ± 0.78^a^	13.31 ± 0.90^e^
C18:1	20.15 ± 0.36^f^	26.62 ± 0.72^d^	19.16 ± 0.96^f^	18.09 ± 0.23^g^	30.92 ± 0.40^c^	34.71 ± 0.56^b^	16.18 ± 0.03^h^	19.75 ± 0.26^f^	26.55 ± 0.46^d^	46.76 ± 1.09^a^	21.37 ± 1.23^e^
C18:2	27.98 ± 0.49^g^	37.48 ± 1.00^e^	28.31 ± 0.19^g^	27.15 ± 0.31^g^	45.09 ± 0.52^c^	51.91 ± 0.90^b^	24.93 ± 0.08^h^	30.84 ± 0.36^f^	43.48 ± 0.18^cd^	68.24 ± 1.34^a^	31.32 ± 1.80^f^
C18:3	1.40 ± 0.03^fg^	2.02 ± 0.13^d^	0.25 ± 0.01^i^	1.37 ± 0.01^g^	2.38 ± 0.01^c^	3.09 ± 0.07^b^	1.16 ± 0.01^h^	1.59 ± 0.02^ef^	1.84 ± 0.37^d^	5.06 ± 0.10^a^	1.63 ± 0.10^e^
C20:0	0.05 ± 0.00^c^	0.08 ± 0.00^b^	0.07 ± 0.00^b^	0.06 ± 0.01^bc^	0.14 ± 0.01^a^	0.03 ± 0.00^d^	0.04 ± 0.01^c^	0.06 ± 0.00^bc^	0.15 ± 0.04^a^	0.01 ± 0.00^d^	0.07 ± 0.01^b^
C20:1	0.46 ± 0.01^cd^	0.63 ± 0.05^ab^	0.53 ± 0.01^bc^	0.40 ± 0.02^d^	0.72 ± 0.01^a^	0.01 ± 0.00^f^	0.25 ± 0.17^e^	0.54 ± 0.01^bc^	0.65 ± 0.11^a^	0.02 ± 0.00^f^	0.53 ± 0.06^bc^
C20:2	0.53 ± 0.01^ef^	0.67 ± 0.04^c^	0.54 ± 0.00^e^	0.54 ± 0.00^e^	0.80 ± 0.02^b^	0.85 ± 0.01^b^	0.45 ± 0.01^f^	0.70 ± 0.01^c^	0.64 ± 0.12^cd^	1.24 ± 0.12^a^	0.56 ± 0.03^de^
C20:3	0.33 ± 0.17^d^	0.20 ± 0.03^d^	0.20 ± 0.02^d^	0.86 ± 0.14^bc^	1.21 ± 0.24^ab^	0.91 ± 0.60^bc^	0.48 ± 0.23^cd^	0.13 ± 0.02^d^	0.17 ± 0.02^d^	1.54 ± 0.23^a^	0.48 ± 0.66^cd^
C20:4	2.74 ± 0.07^ef^	2.86 ± 0.15^e^	2.39 ± 0.01^f^	3.09 ± 0.04^de^	3.95 ± 0.01^b^	3.62 ± 0.08^bc^	3.62 ± 0.02^bc^	3.68 ± 0.07^bc^	3.41 ± 0.66^cd^	4.83 ± 0.08^a^	3.64 ± 0.27^bc^
C22:6n3	0.41 ± 0.01^ab^	0.30 ± 0.01^h^	0.39 ± 0.00^c^	–	0.38 ± 0.02^d^	0.36 ± 0.00^e^	0.35 ± 0.00^ef^	0.38 ± 0.01^cd^	0.34 ± 0.00^f^	0.41 ± 0.01^ab^	0.33 ± 0.01^g^
SFA	24.12 ± 0.29^f^	31.57 ± 0.81^d^	24.54 ± 0.26^f^	23.5 ± 0.24^f^	36.91 ± 0.71^c^	41.41 ± 0.72^b^	20.84 ± 0.06^g^	26.63 ± 0.34^e^	31.63 ± 0.90^d^	55.96 ± 1.38^a^	27.51 ± 0.68^e^
MUFA	23.28 ± 0.41^e^	30.39 ± 0.63^d^	43.91 ± 0.82^b^	21.04 ± 0.25^f^	36.02 ± 0.48^c^	41.58 ± 0.33^b^	18.64 ± 0.04^g^	22.94 ± 0.33^f^	30.09 ± 1.18^d^	56.43 ± 0.58^a^	24.88 ± 0.63^e^
PUFA	31.13 ± 0.56^g^	40.64 ± 1.15^d^	31.09 ± 0.20^g^	30.24 ± 0.35^g^	49.33 ± 0.53^c^	55.89 ± 0.98^b^	28.9 ± 0.10^h^	34.9 ± 0.43^f^	47.23 ± 0.84^c^	73.48 ± 0.42^a^	38.29 ± 1.07^de^
EFA	27.98 ± 0.49^g^	37.48 ± 1.00^e^	28.31 ± 0.19^g^	27.15 ± 0.31^g^	45.09 ± 0.52^c^	51.91 ± 0.90^b^	24.93 ± 0.08^h^	30.84 ± 0.36^f^	43.48 ± 0.18^d^	68.24 ± 0.34^a^	31.32 ± 0.80^f^

Values are mean ± SD (n = 3). Different superscript letters within the same row indicate significant differences among treatments (*p* < 0.05). Treatment codes are defined in Table S1.

### 3.7 Analysis of volatile compounds

A total of 57 volatile compounds were identified across the 11 groups, primarily comprising aldehydes (17) and alcohols (12), followed by ketones (4), hydrocarbons (4), esters (3), and furans (1). The diversity of volatile compounds varied among processing methods, ranging from 21 compounds (SV1) to 41 (M1).

In terms of volatile composition, aldehydes were the dominant class by relative content, followed by alcohols and ketones, consistent with lipid oxidation pathways in poultry meat (Wang et al., 2024). The relatively high abundance of hexanal is consistent with its well-established origin from oxidation of ω-6 PUFAs (notably linoleic acid) (Wu et al., 2023). In the present study, microwave pretreatment generally increased aldehyde abundance compared with sous-vide controls, which is in line with the observed shifts in fatty acid composition after processing (Table 4). Rather than attributing this pattern to a single pathway, we interpret it as the combined outcome of substrate availability (PUFAs) and thermal history that governs lipid oxidation kinetics.

Alcohols, typically formed through decomposition of lipid hydroperoxides, were the second most prominent group (Zhang et al., 2018). Alcohol abundance increased after microwave pretreatment, and the 61 °C/90 min sous-vide condition generally yielded more alcohol compounds than 58 °C/140 min, indicating temperature-dependent conversion among oxidation intermediates. Because aldehydes and alcohols can interconvert and participate in secondary reactions during heating, the observed class-level differences likely reflect the overall balance of formation and consumption during processing rather than a monotonic trend for any single compound (Yang et al., 2019).

**Fig. 2:**
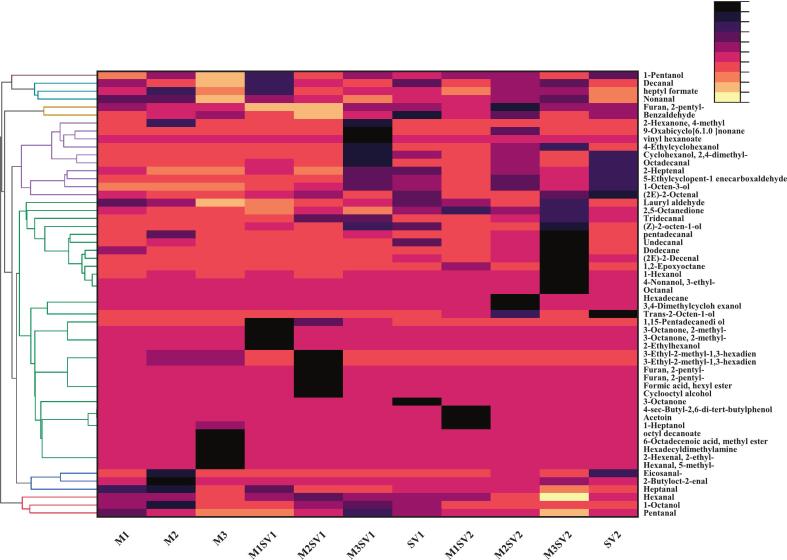
Hierarchical clustering heatmap of volatile compounds in chicken breast samples determined by HS-SPME–GC–MS. Rows represent individual volatiles and columns represent treatment groups. Color intensity indicates normalized relative abundance (scaled within each compound), with darker colors representing higher levels.

**Fig. 3:**
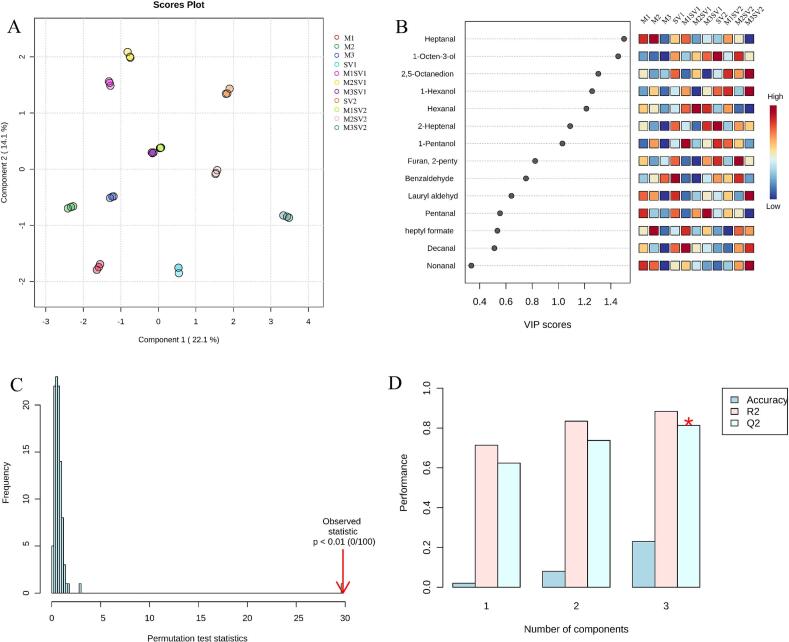
Partial least squares discriminant analysis (PLS-DA) of volatile profiles in chicken breast samples. (A) Score plot showing separation among treatment groups. (B) Variable importance in projection (VIP) scores of discriminant volatiles with the corresponding relative-abundance heatmap (high to low). (C) Permutation test (100 permutations) for model validation. (D) Model performance (accuracy, R^2^, and Q^2^) as a function of the number of latent components.

To further examine treatment-dependent differences, a heatmap based on cluster analysis was constructed (Fig. 2), grouping volatiles into seven clusters. Microwave pretreatment for 40–60 s increased the abundance of several key volatiles relative to sous-vide controls (*p* < 0.05). Importantly, the multivariate model (Fig. 3) showed clear separation among cooking methods, and the VIP-ranked discriminant variables were dominated by aldehydes and alcohols, consistent with the class-level patterns described above. Taken together with the fatty acid results (Table 4), these findings support the interpretation that microwave pretreatment reshaped aroma profiles primarily through lipid-related pathways, while the subsequent sous-vide condition influenced the extent to which these volatiles accumulated or were transformed during heating.

### 3.8 Partial least squares discriminant analysis (PLS-DA)

PLS-DA is a supervised multivariate statistical method used to model the relationship between multiple independent and dependent variables. Compared with principal component analysis (PCA), PLS-DA better highlights variables that contribute to group discrimination (Sun et al., 2024; Zhang et al., 2024). As shown in Fig. 3A, the PLS-DA score plot revealed clear separation between the control samples and the processed groups, and the different treatment groups were also distinguishable, which is consistent with the clustering pattern observed in the heatmap (Fig. 2).

Model reliability was evaluated using permutation testing (Fig. 3C), which supported the robustness of the discrimination, with R^2^(cum) = 0.884 and Q^2^(cum) = 0.813. To identify the volatiles most responsible for group separation, variable importance in projection (VIP) scores were calculated (Xu et al., 2023). As shown in Fig. 3B, seven discriminant compounds were identified as potential aroma markers, primarily comprising aldehydes, alcohols, and ketones, which effectively differentiated chicken breasts processed under different thermal conditions.

### 3.9 Fatty acid oxidation and volatile flavor formation

Fatty acid composition serves as a fundamental substrate for the generation of volatile flavor compounds, particularly through the oxidative degradation of polyunsaturated fatty acids (PUFAs) (Zhao et al., 2024). In the current study, microwave pretreatment significantly modulated the fatty acid profile (Table 4), which was accompanied by a marked increase in lipid-derived volatiles—most notably hexanal, a primary oxidation marker of C18:2 (linoleic acid). This trend suggests an accelerated oxidative conversion of n-6 PUFAs mediated by the intensified thermal energy of the microwave phase. However, the final volatile abundance is likely the result of a kinetic balance between the formation, consumption, and secondary transformation of these intermediates (Sun et al., 2023).

Analysis via HS-SPME–GC–MS identified 57 distinct volatile compounds, with aldehydes (27.3%) and alcohols (22.8%) emerging as the dominant chemical classes. The relative abundance of 2-pentylfuran reached its maximum in the M3SV2 group, aligning with established pathways involving the degradation of linoleic acid hydroperoxides (Wall et al., 2019). Notably, in contrast to traditional high-temperature roasting—which often triggers excessive oxidation and the accumulation of “off-flavor” markers—the MSV process maintained a moderate and desirable volatile intensity. This equilibrium is consistent with the observations of Khalid et al. (2022), indicating that the anaerobic environment inherent to sous-vide cooking restricts oxygen availability. This “oxygen-shielding” effect effectively attenuates the over-oxidation of lipid markers while facilitating the retention of delicate, heat-labile aroma compounds (Yadav et al., 2025; Zhou et al., 2025). Furthermore, the extended sous-vide duration (58 °C, 140 min) appears to further refine the volatile profile. This prolonged thermal exposure likely extends the reaction window for the maturation of lipid-oxidation products into more complex aromatic patterns, a phenomenon reflected in the distinct treatment separation observed in the multivariate analysis (Fig. 3).

### 3.10 *E*-nose analysis

**Fig. 4:**
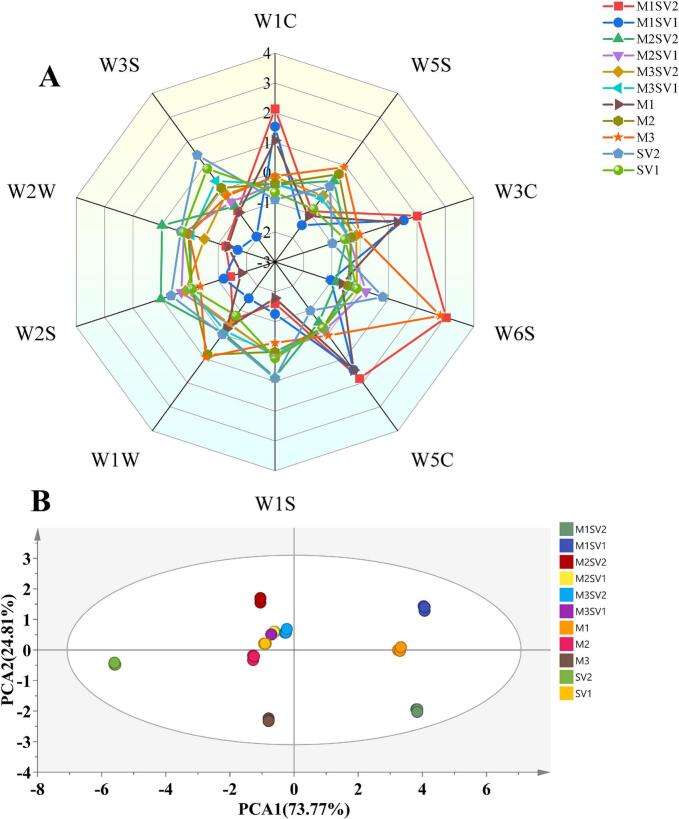
Electronic nose (e-nose) analysis of chicken breast samples processed by different methods. (A) Radar plot of sensor responses. (B) PCA score plot of e-nose data (PC1 = 73.77%, PC2 = 24.81%).

The differences in volatile profiles among the 11 chicken breast samples were analyzed using a PEN 3.5 electronic nose equipped with 10 sensors. The radar plots showed that the sensors responded to volatile odors in the samples (Fig. 4A), indicating that each treatment generated detectable aroma compounds. Compared with the other groups, the microwave-pretreated samples exhibited higher responses for several sensors (e.g., W1C, W3C, W5C, and W6S), suggesting higher overall levels of volatiles associated with aromatic compounds, alkanes, and related components.

PCA of the E-nose data showed that PC1 explained 73.77% of the variance, and the first two principal components collectively accounted for 98.58% (Fig. 4B). The score plot indicated that different microwave pretreatment times and sous-vide conditions produced distinct aroma characteristics. The clustering of M1, M2, M3SV2, M3SV1, and M2SV1 suggests that these treatments generated similar aroma profiles, whereas SV1 and SV2 were separated from the other groups, indicating clear differences in odor patterns.

Because E-nose sensor responses reflect the overall concentration and composition of volatiles (Bojarska et al., 2003), the PCA results were consistent with the GC–MS findings. In particular, samples receiving microwave pretreatment followed by sous-vide cooking showed stronger sensor responses than sous-vide controls, supporting the conclusion that the hybrid process reshaped aroma profiles by increasing key volatile compounds.

## 4 Conclusion

This study demonstrates that microwave pretreatment followed by sous-vide cooking synergistically modulates taste precursors and volatile formation in chicken breast. Two-way ANOVA confirmed a significant interaction between microwave pretreatment time and sous-vide condition, indicating that the combined processing accelerates the accumulation of key umami-related compounds beyond additive effects. Under the optimal combined condition, total free amino acids reached 19.51 ± 0.27 mg g^−1^, with glutamic acid as the dominant taste-active contributor. Umami-enhancing nucleotides also increased substantially, including 5′-GMP [1.82 ± 0.11 mg (100 g)^−1^] and 5′-AMP [2.37 ± 0.15 mg (100 g)^−1^], resulting in higher umami intensity as reflected by EUC and TAV. Volatile analysis showed a 28%–35% increase in major aroma compounds, particularly aldehydes (e.g., hexanal) and alcohols (e.g., 1-octen-3-ol), and multivariate models (PLS-DA and E-nose PCA) confirmed clear aroma discrimination across treatments. Despite these findings, this study has certain limitations. The study's focus on chicken breast muscle suggests that these synergistic effects may vary across meat matrices with different lipid and connective tissue architectures. Further research should integrate protein structural analysis, simulation approaches, and multi-objective optimization to establish scalable processing windows that maximize flavor enhancement while maintaining structural and nutritional integrity required for industrial adoption.

## CRediT authorship contribution statement

**Shuqiang Zhang:** Writing – original draft, Formal analysis, Data curation. **Yungang Cao:** Resources, Investigation, Formal analysis. **Min Li:** Project administration, Investigation, Formal analysis. **Zhijian Wang:** Visualization, Methodology, Funding acquisition. **Bin Yu:** Supervision, Project administration, Methodology, Conceptualization. **Haiteng Tao:** Project administration, Methodology, Investigation. **Wei Gao:** Validation, Supervision, Software. **Jianpeng Li:** Visualization, Validation, Methodology. **Zheng Zhang:** Methodology, Investigation, Funding acquisition. **Haibo Zhao:** Writing – review & editing, Investigation, Funding acquisition. **Zhengzong Wu:** Visualization, Validation, Supervision, Software, Resources. **Bo Cui:** Supervision, Project administration, Investigation.

## Declaration of competing interest

The authors declare that they have no known competing financial interests or personal relationships that could have appeared to influence the work reported in this paper.

## Data Availability

The authors do not have permission to share data.
