# Supplementary material for: Synergistic effects of microwave pretreatment and sous-vide cooking on flavor modulation and taste precursor dynamics in chicken breast
Source: Food Chem X. 2026 May 20;36:104013. doi: 10.1016/j.fochx.2026.104013 (PMC13224382; doi:10.1016/j.fochx.2026.104013)
Supplement: Supplementary file 1 — Supplementary material [file mmc1.docx]

**Table S1** Treatment codes and processing conditions for microwave pretreatment and sous-vide cooking of chicken breast, considering 3 replicates per treatment.

| Treatment group | Pretreatment condition | Main treatment condition |
| --- | --- | --- |
| SV1 | / | 58 ℃, 140 min |
| SV2 | / | 61 ℃, 90 min |
| M1SV1 | 360 W, 40 s | 58 ℃, 140 min |
| M2SV1 | 360 W, 50 s | 58 ℃, 140 min |
| M3SV1 | 360 W, 60 s | 58 ℃, 140 min |
| M1SV2 | 360 W, 40 s | 61 ℃, 90 min |
| M2SV2 | 360 W, 50 s | 61 ℃, 90 min |
| M3SV2 | 360 W, 60 s | 61 ℃, 90 min |
| M1 | 1080 W, 40s | / |
| M2 | 1080 W, 50s | / |
| M3 | 1080 W, 60s | / |

**Table S2** PEN3 electronic nose sensor array and primary selectivity profiles.

| Array number | Sensor Name | Type of substance represented | Performance Description |
| --- | --- | --- | --- |
| 1 | W1C | aromatic | Aromatic ingredients, benzene |
| 2 | W5S | broadrange | High sensitivity, very sensitive to nitrogen oxides |
| 3 | W3C | aromatic | Aromatic component sensitivity, ammonia |
| 4 | W6S | hydrogen | Selective mainly for hydrides |
| 5 | W5C | arom-aliph | Short-chain alkane aromatic components |
| 6 | W1S | broad-methane | Sensitive to methyl groups |
| 7 | W1W | sulphur-organic | Sensitive to sulfides |
| 8 | W2S | broad-alcohol | Sensitive to alcohols, aldehydes and ketones |
| 9 | W2W | sulph-chlor | Aromatic components, sensitive to organic sulfides |
| 10 | W3S | methane-aliph | Sensitive to long chain alkanes |

**Table S3** Two-way ANOVA results for the effects of microwave pretreatment time and sous-vide condition on total FAA content and equivalent umami concentration (EUC). ***P* < 0.01.

|  | Total FAA | EUC |
| --- | --- | --- |
| SV | ** | ** |
| MV | ** | ** |
| MSV | ** | ** |

Note: ** indicates a highly significant difference at *p* < 0.01.

**Fig. S1.** Pearson correlation heatmap showing associations between processing parameters (microwave pretreatment time and sous-vide condition/temperature) and flavor-related indicators, including umami-related nucleotides, free amino acids, and selected key volatile compounds. Color intensity denotes the correlation coefficient (r), with positive and negative correlations indicated by warm and cool colors, respectively. Asterisks indicate statistical significance (**P* < 0.05).
